# Complete genome sequence of *Pseudomonas asiatica* RYYT.1b, a glyphosate-resistant strain isolated from Hong Kong soil

**DOI:** 10.1128/mra.00232-25

**Published:** 2025-06-10

**Authors:** R. Y. Y. Tang, K. M. Leung, G. K. K. Lai, S. D. J. Griffin

**Affiliations:** 1 Shuyuan Molecular Biology Laboratory, The Independent Schools Foundation Academy, Hong Kong, China; The University of Arizona, Tucson, Arizona, USA

**Keywords:** *Pseudomonas asiatica*, glyphosate, phosphate-solubilizing, bioremediation

## Abstract

The complete genome of *Pseudomonas asiatica* RYYT.1b, a glyphosate-resistant strain from Hong Kong soil, was established through hybrid assembly. It has a single circular chromosome, with a genome size of 6,102,102 bp (63.02% G + C).

## ANNOUNCEMENT

*Pseudomonas asiatica* is a Gram-negative, oxidase-positive, fluorescent pseudomonad of the *Pseudomonas putida* group commonly found in soil. First identified in a clinical setting (1), it is noted for its tolerance of extreme conditions and metabolic versatility (2, 3). Utilizing a wide range of carbon sources, it lends itself to bioremediation and industrial biosynthesis (4, 5). *P. asiatica* JP233 demonstrated phosphate-solubilizing ability and plant-growth promotion (6), and here, RYYT.1b was isolated from urban soil in Pokfulam, Hong Kong (22.2652601N, 114.1299767E; 11 October 2022) as a strain resistant to glyphosate (*N*-[phosphonomethyl]glycine), the shikimate pathway-inhibiting herbicide (7).

Soil (0.5 g; collected at a depth of about 5 cm from an area not previously treated with glyphosate) was vortexed with 24.5 mL saline (0.9% [wt/vol]) and the mixture allowed to settle for 5 minutes. A total of 50 µL of the clearer supernatant and 0.2 mL of 0.085% (wt/vol) aqueous glyphosate were added to 1.8 mL Luria broth (8), and the mixture was incubated overnight at room temperature. After a 5,000-fold dilution with saline, 100 µL of the culture was spread onto Luria agar for overnight incubation. A resultant colony of RYYT.1b was passaged to purity on Luria agar and confirmed to grow on modified Pikovskaya’s agar (9) containing 2% (wt/vol) glyphosate as the sole source of phosphate. A single colony of RYYT.1b was streaked onto Luria agar for overnight growth before harvesting growth from the plate for DNA extraction (DNeasy PowerSoil Pro Kit, Qiagen GmbH). All agar incubations were at 27°C.

Paired-end short-read libraries (NEBNext Ultra DNA Library Prep Kit, Illumina, Inc.) were sequenced via the NovaSeq 6000 platform using an SP PE250 flowcell and v1.5 Reagent Kit. A total of 6,138,448 raw reads (mean length 250 bp; SRX27106818) were quality-filtered and trimmed using TrimGalore! v0.6.7 (stringency:3; -e:0.2), producing 3,064,557 read pairs (mean length 247 bp, total ~754 Mbp). Long-read libraries, prepared from the same extracted DNA using the Rapid Barcoding Kit SQK-RBK004 without size selection, were sequenced via Oxford Nanopore’s Spot-ON Flow Cell (vR9.4.1), MinION sequencer, and MinKNOW v20.06.2 software, with base-calling by Guppy v6.1.55 (high-accuracy mode). The final long-read data set of 193,683 raw reads (mean length 5,136 bp; SRX27106819) was trimmed by Filtlong v0.2.1 (min_length: 2,000; target_bases: 500 Mbp; length_weight: 10), selecting 104,718 reads (mean length 8,549 bp, N50 11,245; total ~748.1 Mbp) to scaffold the assembly. Default parameters were used for all software unless otherwise specified.

Illumina and MinION data sets were combined using Unicycler v0.4.8-beta (10) yielding a single circular chromosome (6,102,102 bp, 63.02% G + C; rotated to begin *dnaA* [B1J3Y2]) with mean coverage 148× (judged 100% complete with 0.35% contamination by CheckM v1.2.2 [11]), which was submitted to NCBI PGAP v6.5 (12) for annotation. RYYT.1b shares an average nucleotide identity (ANIu by EZBioCloud [13]) of 97.87% with *P. asiatica* C-B8A (CP157874).

Chromosomal genes (predicted by NCBI PGAP v6.5 or found by BLAST in Uniprot [14]) linked to phosphate solubilization and/or glyphosate degradation are shown in Table 1.

**TABLE 1:** Predicted genes in RYYT.1b linked to phosphate solubilization and/or glyphosate degradation^*a*^

Gene(s)	Locus (NCBI)	Activity	Reference
*pqqFABCDE* *pqqD* *pqqA* *pqqE*	QQ994_01810 to QQ994_01835 QQ994_16455 QQ994_16460 QQ994_01770	Pyrroloquinoline quinone coenzyme (PQQ) biosynthesis	(15)
*gcd*	QQ994_22110	PQQ-dependent glucose dehydrogenase (EC 1.1.5.2)	(16)
*gadh3-gadh1-gadh2* (*three isoforms*)	QQ994_10425 to QQ994_10415 QQ994_10470 to QQ994_10480 QQ994_15070 to QQ994_15060	Gluconate dehydrogenase	(6)
*pitA* *pitB*	QQ994_18195 QQ994_22580	Inorganic phosphate transporter	(16)
*phoB* *phoR* *phoU*	QQ994_27150 QQ994_27155 QQ994_27175	Regulation of phosphate transport	(17)
*pstSCAB*	QQ994_16670 to QQ994_16655	Phosphate-specific transporter	(16)
*ugpABCE*	QQ994_05075	sn-glycerol-3-phosphate ABC transporter	(16)
*ptxS-kguEKTD*	QQ994_15055 to QQ994_15035	2-ketogluconate utilization	(18)
*phnA* *phnB* *phnC* *phnE* *phnR* *phnN*	QQ994_03475 QQ994_15370 QQ994_04330 QQ994_04340 QQ994_06370 QQ994_19780	Phosphonate degradation	(19)
*soxBDAG* (two copies)	QQ994_01535 to QQ994_01550 QQ994_11460 to QQ994_11475	Sarcosine oxidase	(20)

^*a*^ Predicted by NCBI PGAP v6.5 (12) or found by BLAST in Uniprot (14).

## Data Availability

Sequencing data for *Pseudomonas asiatica* RYYT.1b are available through NCBI under GenBank assembly GCA_030291675 and GenBank accession CP127872. Raw reads may be found under SRX27106818 (NovaSeq) and SRX27106819 (MinION).

## References

[1] Tohya M, Watanabe S, Teramoto K, Shimojima M, Tada T, Kuwahara-Arai K, War MW, Mya S, Tin HH, Kirikae T. 2019. Pseudomonas juntendi sp. nov., isolated from patients in Japan and Myanmar. Int J Syst Evol Microbiol 69:3377–3384. doi:10.1099/ijsem.0.003623 31368883

[2] Volke DC, Calero P, Nikel PI. 2020. Pseudomonas putida. Trends Microbiol 28:512–513. doi:10.1016/j.tim.2020.02.015 32396829

[3] Mozejko-Ciesielska J. 2021. Chapter 10, *Pseudomonas putida*–based cell factories, p 165–181. In Singh V (ed), Microbial cell factories engineering for production of biomolecules

[4] Hester KL, Luo J, Sokatch JR. 2000. Purification of Pseudomonas putida branched-chain keto acid dehydrogenase E1 component. Methods Enzymol 324:129–138. doi:10.1016/s0076-6879(00)24226-2 10989425

[5] Thi Nguyen T, Lama S, Kumar Ainala S, Sankaranarayanan M, Singh Chauhan A, Rae Kim J, Park S. 2021. Development of Pseudomonas asiatica as a host for the production of 3-hydroxypropionic acid from glycerol. Bioresour Technol 329:124867. doi:10.1016/j.biortech.2021.124867 33640696

[6] Wang L, Zhou F, Zhou J, Harvey PR, Yu H, Zhang G, Zhang X. 2022. Genomic analysis of Pseudomonas asiatica JP233: an efficient phosphate-solubilizing bacterium. Genes 13:2290. doi:10.3390/genes13122290 36553557 PMC9777792

[7] van Bruggen AHC, Finckh MR, He M, Ritsema CJ, Harkes P, Knuth D, Geissen V. 2021. Indirect effects of the herbicide glyphosate on plant, animal and human health through its effects on microbial communities. Front Environ Sci 9. doi:10.3389/fenvs.2021.763917

[8] MacWilliams MP, Liao MK. 2006. Luria broth (LB) and Luria agar (LA) media and their uses protocol. ASM MicrobeLibrary. American Society for Microbiology. Available from: https://asm.org/Protocols/Luria-Broth-LB-and-Luria-Agar-LA-Media-and-Their-U

[9] Pikovskaya RI. 1948. Mobilisation of phosphorus in soil in connection with vital activity of some microbial species. Microbiologiya 17:362–370.

[10] Wick RR, Judd LM, Gorrie CL, Holt KE. 2017. Unicycler: resolving bacterial genome assemblies from short and long sequencing reads. PLoS Comput Biol 13:e1005595. doi:10.1371/journal.pcbi.1005595 28594827 PMC5481147

[11] Parks DH, Imelfort M, Skennerton CT, Hugenholtz P, Tyson GW. 2015. CheckM: assessing the quality of microbial genomes recovered from isolates, single cells, and metagenomes. Genome Res 25:1043–1055. doi:10.1101/gr.186072.114 25977477 PMC4484387

[12] Haft DH, DiCuccio M, Badretdin A, Brover V, Chetvernin V, O’Neill K, Li W, Chitsaz F, Derbyshire MK, Gonzales NR, Gwadz M, Lu F, Marchler GH, Song JS, Thanki N, Yamashita RA, Zheng C, Thibaud-Nissen F, Geer LY, Marchler-Bauer A, Pruitt KD. 2018. RefSeq: an update on prokaryotic genome annotation and curation. Nucleic Acids Res 46:D851–D860. doi:10.1093/nar/gkx1068 29112715 PMC5753331

[13] Chalita M, Kim YO, Park S, Oh HS, Cho JH, Moon J, Baek N, Moon C, Lee K, Yang J, Nam GG, Jung Y, Na SI, Bailey MJ, Chun J. 2024. EzBioCloud: a genome-driven database and platform for microbiome identification and discovery. Int J Syst Evol Microbiol 74:006421. doi:10.1099/ijsem.0.006421 38888585 PMC11261700

[14] Bateman A, Martin M-J, Orchard S, Magrane M, Adesina A, Ahmad S, Bowler-Barnett EH, Bye-A-Jee H, Carpentier D, Denny P, et al.. 2025. UniProt: the universal protein knowledgebase in 2025. Nucleic Acids Res 53:D609–D617. doi:10.1093/nar/gkae1010 39552041 PMC11701636

[15] Oteino N, Lally RD, Kiwanuka S, Lloyd A, Ryan D, Germaine KJ, Dowling DN. 2015. Plant growth promotion induced by phosphate solubilizing endophytic Pseudomonas isolates. Front Microbiol 6:745. doi:10.3389/fmicb.2015.00745 26257721 PMC4510416

[16] Wu X, Rensing C, Han D, Xiao KQ, Dai Y, Tang Z, Liesack W, Peng J, Cui Z, Zhang F. 2022. Genome-resolved metagenomics reveals distinct phosphorus acquisition strategies between soil microbiomes. mSystems 7:e0110721. doi:10.1128/msystems.01107-21 35014868 PMC8751388

[17] Pan L, Cai B. 2023. Phosphate-solubilizing bacteria: advances in their physiology, molecular mechanisms and microbial community effects. Microorganisms 11:2904. doi:10.3390/microorganisms11122904 38138048 PMC10745930

[18] Sun L, Yang W, Li L, Wang D, Zan X, Cui F, Qi X, Sun L, Sun W. 2024. Characterization and transcriptional regulation of the 2-ketogluconate utilization operon in Pseudomonas plecoglossicida. Microorganisms 12:2530. doi:10.3390/microorganisms12122530 39770733 PMC11678583

[19] Hove-Jensen B, Zechel DL, Jochimsen B. 2014. Utilization of glyphosate as phosphate source: biochemistry and genetics of bacterial carbon-phosphorus lyase. Microbiol Mol Biol Rev 78:176–197. doi:10.1128/MMBR.00040-13 24600043 PMC3957732

[20] Willsey GG, Wargo MJ. 2016. Sarcosine catabolism in Pseudomonas aeruginosa is transcriptionally regulated by SouR. J Bacteriol 198:301–310. doi:10.1128/JB.00739-15 26503852 PMC4751790

